# Oridonin synergistically enhances JQ1-triggered apoptosis in hepatocellular cancer cells through mitochondrial pathway

**DOI:** 10.18632/oncotarget.21880

**Published:** 2017-10-16

**Authors:** Hua-Peng Zhang, Gong-Quan Li, Wen-Zhi Guo, Guang-Hui Chen, Hong-Wei Tang, Bing Yan, Jie Li, Jia-Kai Zhang, Pei-Hao Wen, Zhi-Hui Wang, Jian-Feng Lv, Shui-Jun Zhang

**Affiliations:** ^1^ Department of Hepatobiliary and Pancreatic Surgery, The First Affiliated Hospital of Zhengzhou University, Zhengzhou, Henan, China; ^2^ Open and Key Laboratory of Hepatobiliary & Pancreatic Surgery and Digestive Organ Transplantation at Henan Universities, The First Affiliated Hospital of Zhengzhou University, Zhengzhou, Henan, China; ^3^ Zhengzhou Key Laboratory of Hepatobiliary & Pancreatic Diseases and Organ Transplantation, Zhengzhou, Henan, China

**Keywords:** JQ1, Oridonin, HCC, Apoptosis, Bcl-2

## Abstract

Bromodomain and Extra-Terminal Domain (BET) inhibitors, such as JQ1 have emerged as novel drug candidates and are being enthusiastically pursued in clinical trials for the treatment of cancer. However, many solid cancers are resistance to BET inhibitors. To explore methods for improving the therapeutic potential of BET inhibitors, we investigated the combinational activity of JQ1 with Oridonin, a bioactive molecules derived from Traditional Chinese Medicine in hepatocellular carcinoma (HCC) cells. Our results showed that Oridonin synergistically enhanced the abilities of JQ1 to inhibit cell viability in HCC cells and, significantly augmented JQ1-triggered apoptosis in HCC cells and in HCC cancer stem-like cells. Moreover, Oridonin dose-dependently inhibited the expression of several anti-apoptotic proteins, such as Bcl-2, Mcl-1, and x-linked inhibitor of apoptosis (xIAP) in HCC cells. Cell fractionation and western blotting analysis showed that the enhancement of apoptosis by Oridonin was associated with cytochrome c release, activation of caspase-9, -3 and cleavage of PARP, indicating the activation of mitochondrial apoptosis pathway. Altogether, our findings demonstrate that Oridonin may be used to effectively enhance the sensitivity of BET inhibitors in HCC therapy via downregulation of the expression of multiple anti-apoptotic proteins.

## INTRODUCTION

Hepatocellular cancer (HCC) is one of the most aggressive cancers and is the leading cause of cancer-related mortality in China [1]. Surgical resection and liver transplantation are suitable in only a very small fraction of HCC patients, while most patients required chemotherapy. However, conventional chemotherapy is often ineffective in HCC patients. Therefore, it is imperative to explore novel therapeutic methods for this lethal disease [1].

Bromodomain and extra-terminal (BET) proteins are epigenetic readers of acetylated histones, which play an important role in transcription of genes involved in cell cycle regulation and apoptosis. Epigenetic dysfunction caused by aberrantly high expression of BET genes has been considered as a critical factor for cancerogenesis and cancer progression in both blood and solid cancers [2]. Therefore, targeting BET proteins with small molecule BET inhibitors, such as JQ1 has become an attractive novel cancer therapeutic strategy [2]. Previously we and others showed that BET inhibitors had anticancer activity in HCC and many other types of cancers, suggesting that these BET inhibitors hold promise for cancer patients [3–5]. Nevertheless, BET inhibitors generally have limited efficacy in advanced solid cancers, and do not induce cancer remission in tumor-bearing mice in most preclinical tumor models [6–8]. Moreover, several studies have indicated that BET inhibitors only trigger feeble or modest apoptosis in solid tumor cells [6–10]. These findings suggest that solid cancers are intrinsic resistance, and/or acquired resistance to monotherapy of BET inhibitors, posing a serious problem for the potential clinical utility of this kid of new drugs in the future [6–10].

Oridonin is an active diterpenoid isolated from Rabdosia rubescens which has been widely used to treat tumors and other conditions in traditional Chinese Medicine for a very long time. Recent studies revealed that Oridonin potently inhibited the expression of a panel of oncogenes, such as Bcl-2 family proteins, inhibitors of apoptosis (IAPs) family proteins and the activity of nuclear factor-KB (Nf-kB); thus it can elicit strong anti-tumor activities in many human cancers in clinic. Oridonin has also demonstrated synergistic effect with various anticancer agents by interfering cell proliferation signaling and/or promoting apoptosis signaling pathway [11, 12]. In this study, we investigated whether Oridonin could be used to overcome JQ1-resistance in HCC cells. Our data suggested that through inhibiting the expression of Bcl-2, Mcl-1 and x-linked inhibitor of apoptosis (xIAP), Oridonin synergistically enhanced the anti-HCC activity of JQ1 through mitochondrial pathway.

## RESULTS

### Oridonin inhibits Bcl-2, Mcl-1 and xIAP expression in HCC cells

**Figure 1:**
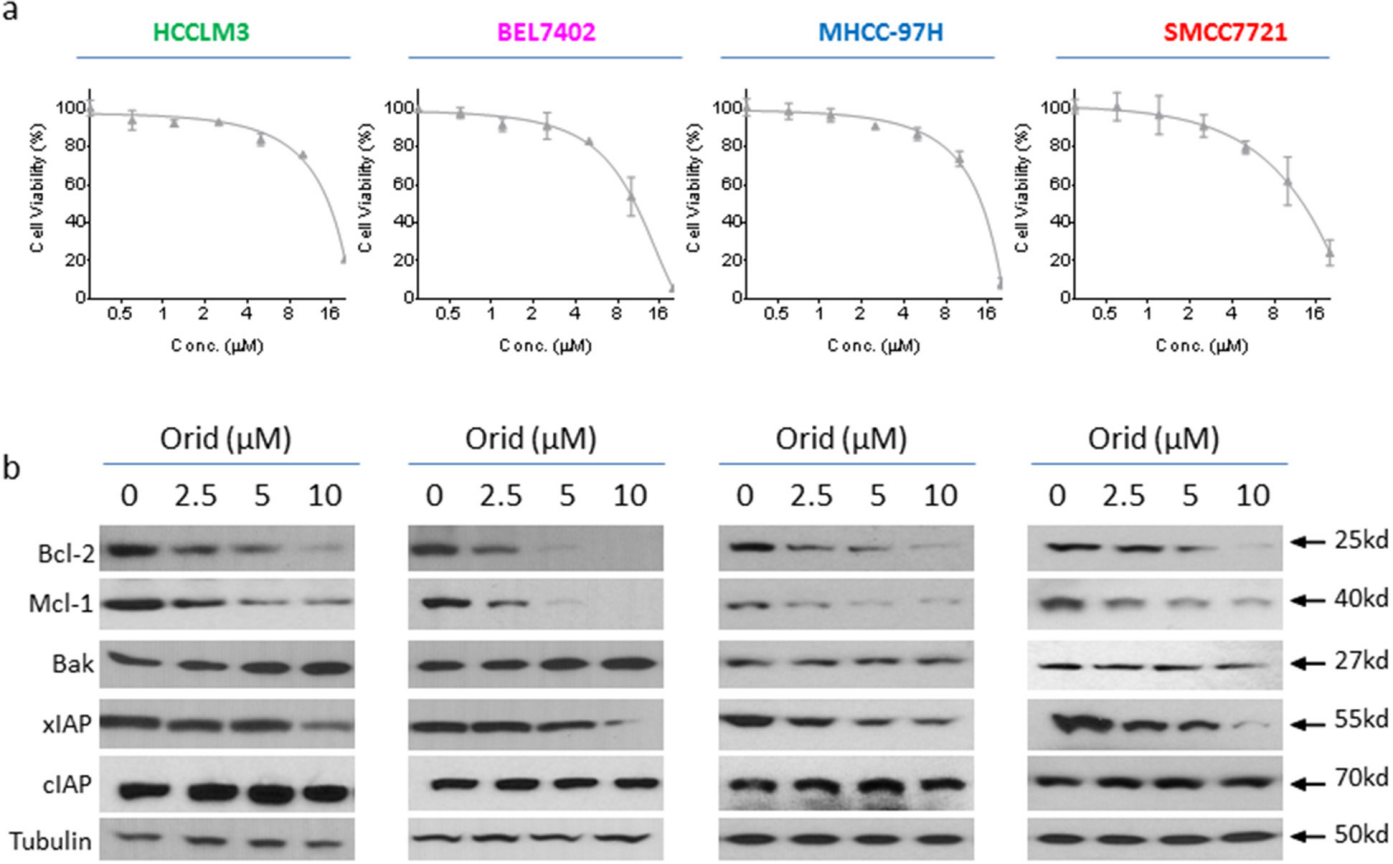
Oridonin inhibits the expression of multiple anti-apoptotic proteins in HCC cell lines HCC HCCLM3, BEL7402, MHCC-97H and SMMC7721 cell lines were treated by Oridonin for 24 h. **(a)** cell viability was determined by CCK-8 assay. The data are representative results of three independent experiments. **(b)** Cells were harvested and cell lysates were examined for the expression of Bcl-2, Mcl-1, Bak, xIAP, cIAP-1/2. Tubulin was used as a loading control.

Previous studies showed that Oridonin was able to modulate the expression of apoptosis-related proteins in other cancer cells [11, 12]; we here investigated if Oridonin had a similar effect in HCC cells. Firstly, we examined the inhibitory effect of Oridonin alone on the cell viability in a panel of 4 HCC cell lines that include HCCLM3, BEL7402, MHCC-97H and SMMC7721 cell lines. We found that treatment with Oridonin at 2.5 - 10 μM for 24 h partially inhibited cell viability in these HCC cell lines (Figure 1a). We then treated HCC HCCLM3, BEL7402, MHCC-97H and SMMC7721 cell lines with this concentration-range for 24 h, and then analyzed the alteration of a panel of apoptosis-related proteins with western blotting analysis. The results showed that Oridonin profoundly and dose-dependently reduced protein level of Bcl-2 and Mcl-1, as well as xIAP in all 4 HCC cell lines. In contrast, Oridonin had minimal effect on the levels of Bak and cIAP-1/2 (Figure 1b). These results suggest that Oridonin effectively inhibits the expression of multiple anti-apoptosis proteins in HCC cells.

### Oridonin enhances JQ1-mediated apoptosis in HCC cells

**Figure 2:**
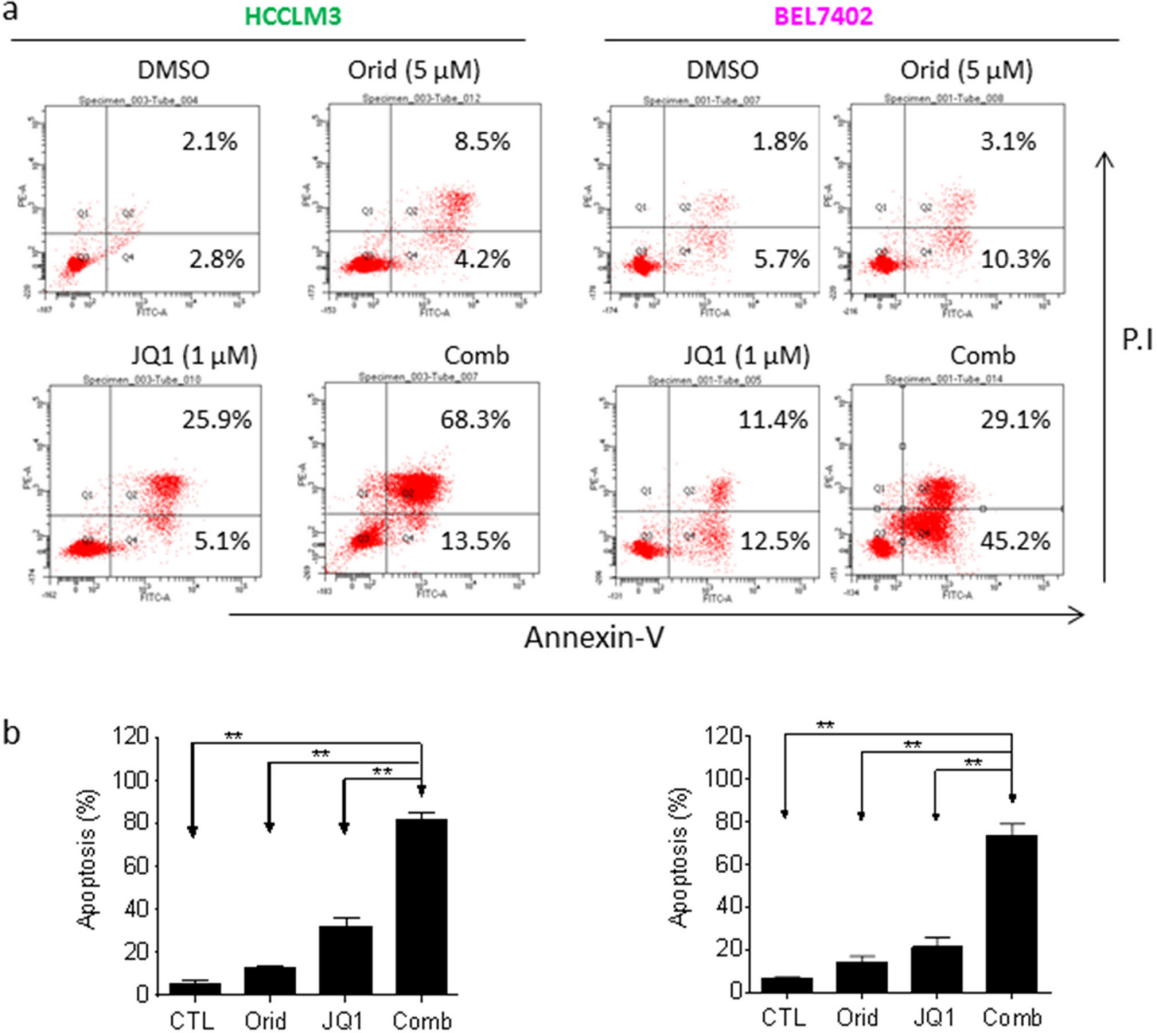
Oridonin enhances JQ1-mediated apoptosis in HCC cells HCC HCCLM3 and BEL7402 cell lines were treated by Oridonin (Orid) alone, JQ1 alone, or both (Comb) for 48 h. Apoptosis was determined by Annxin-V-FITC/PI staining and flow cytometry assay. **(a)** A representative figure was shown for each treatment. **(b)** Average results of three independent experiments were plotted. ^*^, p<0.05, ^* *^, p<0.01.

Apoptosis is the key mechanism by which cancer cells were killed by a variety of therapies [13, 14]. Previously, we noted that JQ1 even at a high concentration (2.5 μM) only had modest apoptotic effect in HCC cells [5]. We here investigated whether Oridonin could enhance JQ1-triggered apoptosis in HCC cells. We treated HCC cell lines with Oridonin alone, JQ1 alone or their combination for 48 h, and then stained with Annexin V-FITC (fluorescein isothiocyanate conjugated) and propidium iodide (PI). We found that in all 4 cell lines, treatment with Oridonin alone at 5 μM did not dramatically increase the percentage of Annexin V-FITC(+) cell subpopulation, and treatment with JQ1 alone at 1 μM increase the percentage of Annexin V-FITC(+) cell subpopulation by 26.2% and 16.5%, respectively, in HCCLM3 and BEL7402 cell lines. In striking contrast, their combination treatment resulted in 51.1% and 49.7% cells positively stained with Annexin V-FITC, respectively, in the two cell lines (Figure 2a, 2b). These results suggest that Oridonin strongly enhances JQ1-triggered apoptosis in HCC cells.

**Figure 3:**
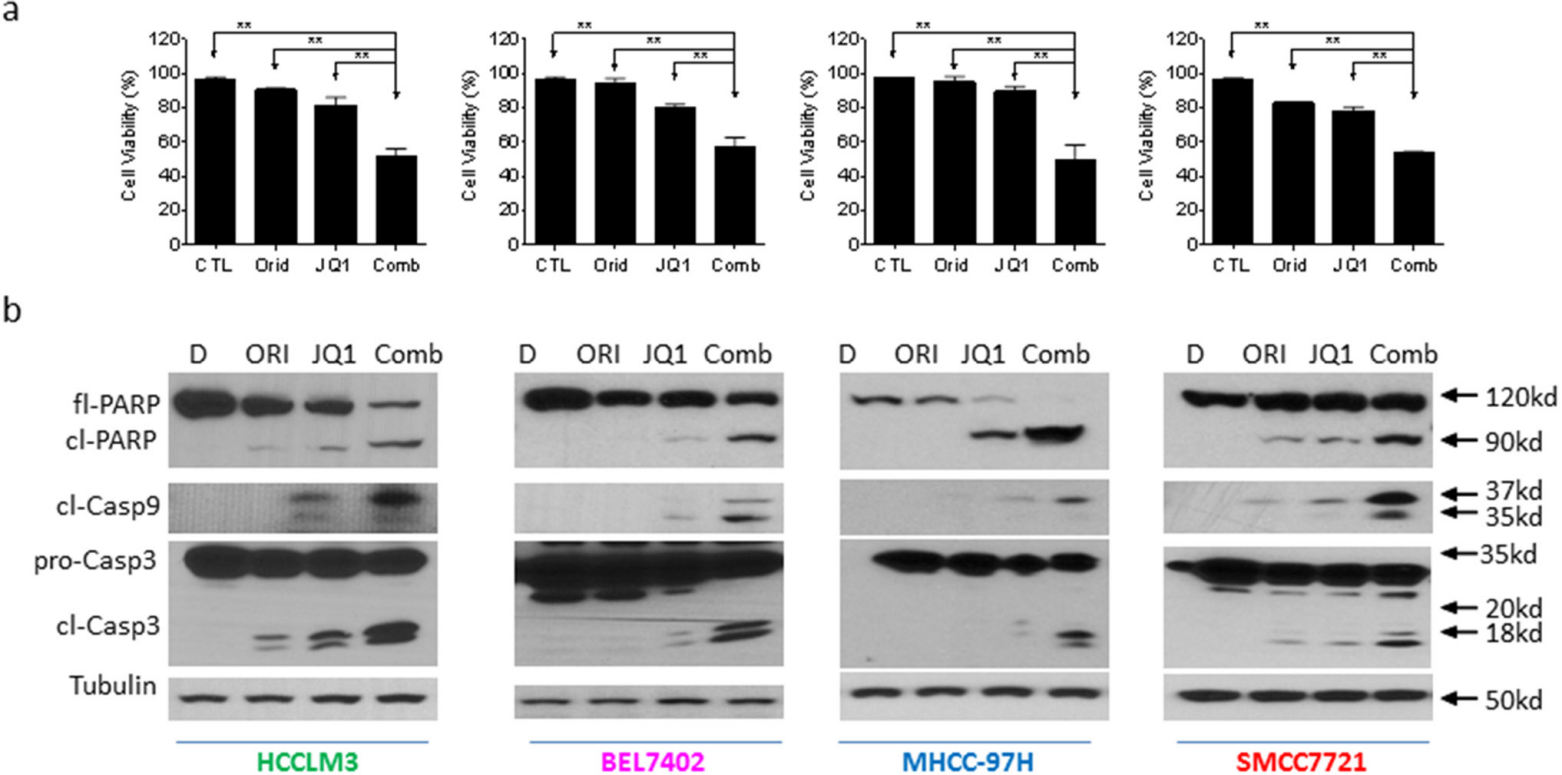
Oridonin enhances JQ1-mediated apoptosis signaling in HCC cells HCC HCCLM3, BEL7402, MHCC-97H and SMMC7721 cell lines were treated by Oridonin (Orid) alone, JQ1 alone, or both (Comb) for 48 h. **(a)** Cell viability was examined by trypan exclusion assay and percentage of viable cells was plotted. **(b)** Cells were harvested and cell lysates were examined for the expression of full-length PARP (fl-PARP), cleaved PARP (cl-PARP), cleaved Caspase-9 (cl-Casp9) and full-length caspase-3 (Fl-Casp3) and cleaved Caspase-3 (cl-Casp3). Tubulin was used as a loading control. The data are representative results of three independent experiments.

Cell viability assay further demonstrated that the combination displayed much stronger ability in inhibition of cell viability in all 4 cell lines (Figure 3a). This was consistent with results of apoptosis assay.

### Oridonin enhances JQ1-triggered mitochondrial apoptosis signaling in HCC cells

We next examined the activation of apoptosis signaling in 4 HCC cell lines treated by Oridonin alone, JQ1 alone or their combination. Western blotting analysis showed that treatment with 5 μM Oridonin for 48 h did not trigger caspase-9 activation and PARP cleavage. Treatment with 1 μM JQ1 for 48 h induced accumulation of cleaved caspases-9, -3. Consistently, JQ1 treatment resulted in modest cleavage of PARP accompanied by reduction of full-length of PARP level. In striking contrast, the combination not only induced a large amount of accumulation of cleaved caspases-9, -3 and cleaved PARP, but also almost completely eliminated the level of full-length PARP (Figure 3b).

**Figure 4:**
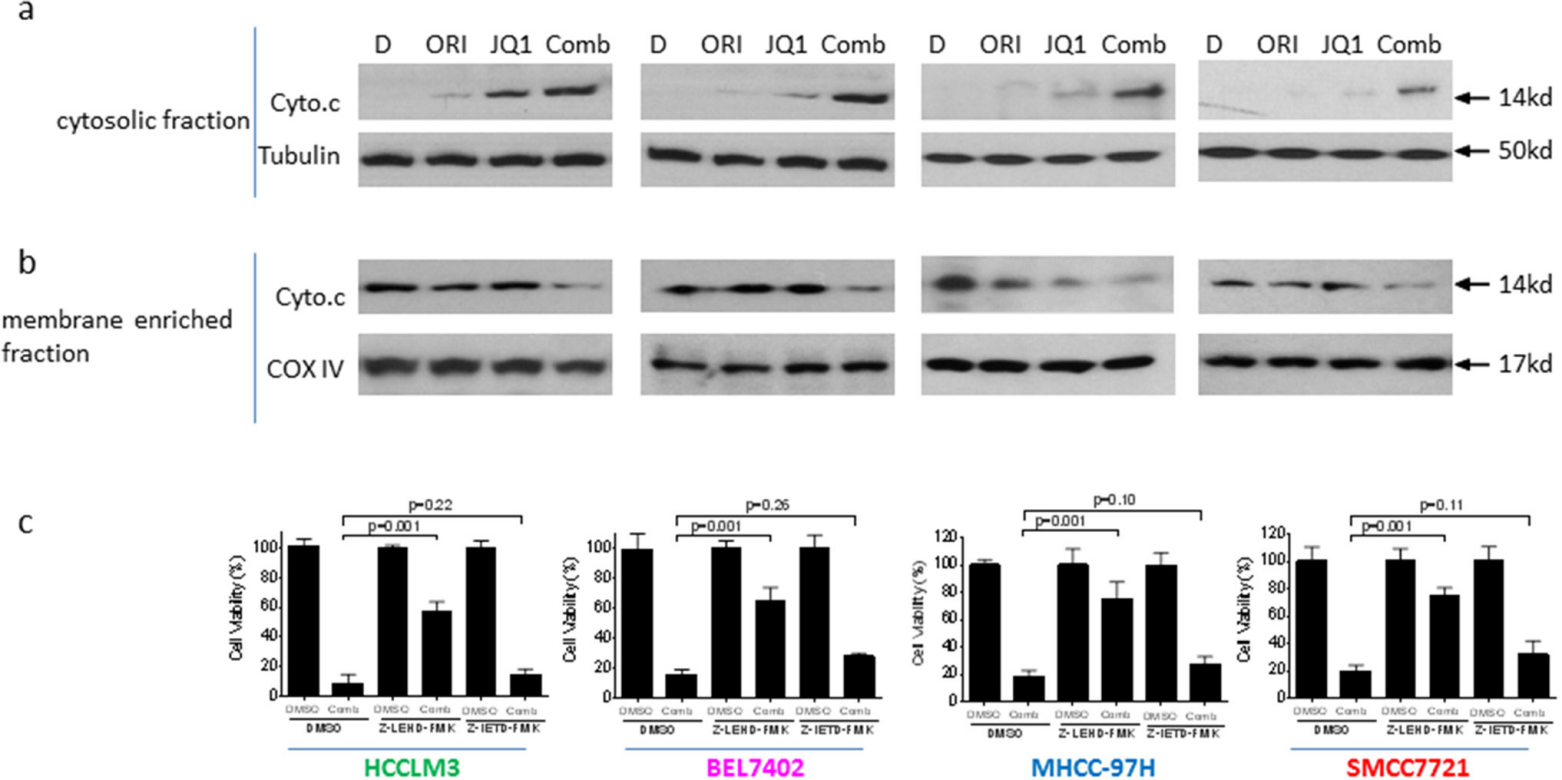
Mitochondrial pathway plays an essential role in the anticancer activity by JQ1 in combination with Oridonin in HCC cells HCC HCCLM3, BEL7402, MHCC-97H and SMMC7721 cell lines were treated by Oridonin (Orid) alone, JQ1 alone, or both (Comb) for 48 h. Cell fractionation were performed as described in the method. The expression of cytochrome c (cyto.c) in cytosolic fraction **(a)** and membrane enriched fraction **(b)** was detected by western blotting analysis. Tubulin or COX IV was used as loading controls. **(c)** HCC HCCLM3, BEL7402, MHCC-97H and SMMC7721 cell lines pretreated with 50 μM caspase-9 inhibitor Z-LEHD-FMK or caspase-8 inhibitor Z-IEHD-FMK for 1 h were further treated by the combination for 48 h, cell death induction was determined with trypan blue exclusion assays. The data are average results of three independent experiments.

To investigate whether mitochondrial pathway was involved in the apoptosis by the combination, we performed subcellular fractionations in 4 HCC cells treated with DMSO, single agents or the combination. We found that the level of cytochrome c was markedly increased in cytosolic fraction of HCC cells treated by the combination, accompanied by reduction of their levels in membrane-fraction (Figure 4a, 4b). These results suggest that the combination treatment causes damage of mitochondrial membrane integrity, which leads to proapoptogenic cytochrome c released into cytoplasm in HCC cells.

To further investigate the role of mitochondrial pathway in the combination, we pretreated the cells with caspase-9 inhibitor Z-LEHD-FMK or caspase-8 inhibitor Z-IETD-FMK, followed by treatment of the combination. We found that suppressing the activity of caspase-9, the initiator caspase for mitochondrial pathway significantly inhibited cell death induction by the combination. However, inhibition of caspase-8, the initiator caspase for death-receptor pathway had no significant effect on the activity of the combination. This rescue experiment suggests that mitochondrial pathway is essential for the apoptosis induction (Figure 4c).

Altogether, these results suggest that Oridonin overcomes resistance of HCC cells to JQ1-mediated apoptosis through mitochondrial signaling pathway.

### Oridonin and JQ1 acts synergistically in inhibition of cell viability in HCC cell lines

**Figure 5:**
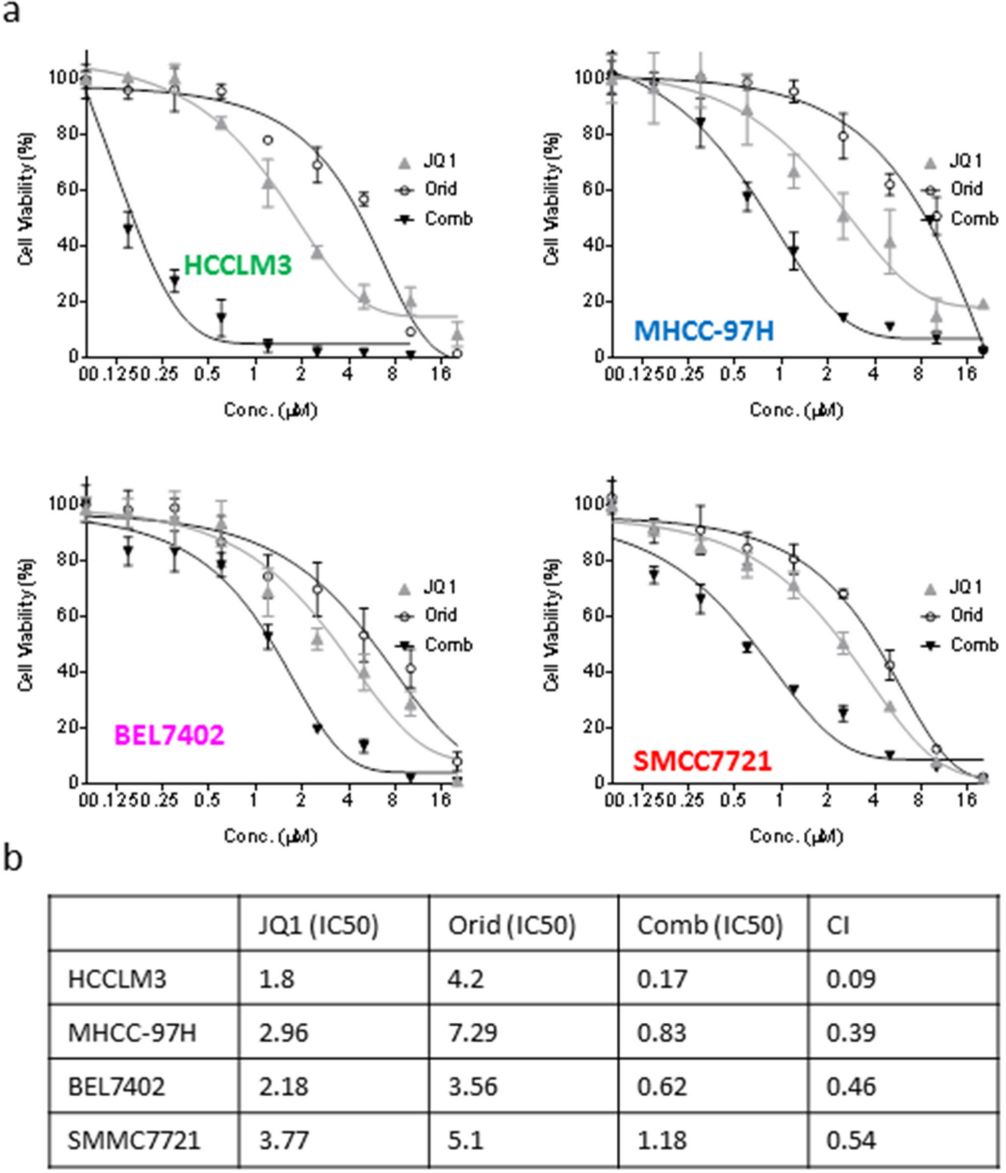
Oridonin acts synergistically with JQ1 in inhibiting cell viability of HCC cells HCC HCCLM3, BEL7402, MHCC-97H and SMMC7721 cell lines were treated by Oridonin alone, JQ1 alone, or both for 120 h. **(a)** cell viability was determined by CCK-8 assay. The data are representative results of three independent experiments. **(b)** The CI was calculated with an equation described in the Material and Method.

In order to investigate whether the enhancement of apoptotic effect could lead to better long-term activity by the combination, we treated the HCC cells with Oridonin alone, JQ1 alone or their combination for 5 days. CCK-8 assays showed that either JQ1 or Oridonin alone dose-dependently inhibited cell viability, and achieved IC50 of 1.8, 4.2 μM, respectively, in HCCLM3 cell line. In striking contrast, their combination had IC50 of 0.17 μM, reducing by 10.6 and 24.7 folds as compared to the IC50 values of either JQ1 or Oridonin alone (Figure 5a). Similarly, the two agents also have strong combination activity in BEL7402, SMMC7721 and MHCC-97H cell lines (Figure 5a). Combinational index values are 0.09, 0.39, 0.46,0.54 μM for HCCLM3, MHCC-97H, BEL7402, SMMC7721 cell lines, respectively (Figure 5b). These results suggest that the two agents have synergistic effect in all these four HCC cell lines.

### The combination of JQ1 with Oridonin potently inhibits the growth of cancer stem-like cells in HCC cell lines

**Figure 6:**
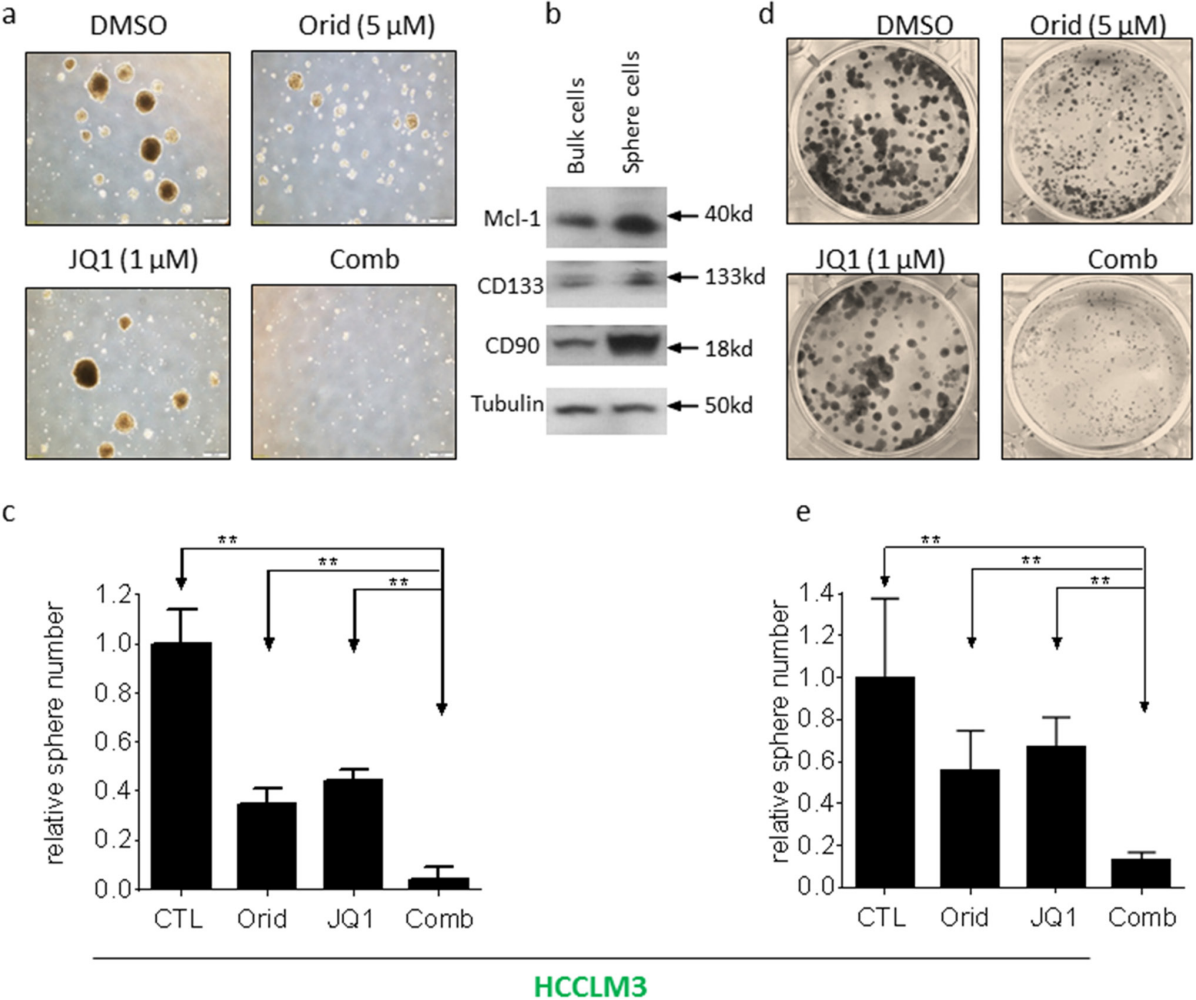
Oridonin significantly enhance JQ1-mediated inhibition of HCCLM3 CSC-like cells growth HCC HCCLM3 cell line was plated in stem cell conditioned culture system allowed for sphere forming. The number of spheres was counted under a microscope and photographed. **(a)** A representative figure was shown for each treatment. **(b)** The expression of CD90, CD133 and Mcl-1 in both bulk cells and sphere cells were examined by western blotting analysis. Tubulin was used as a loading control. **(c)** Average results of three independent experiments were plotted. **(d, e)** HCCLM3 cell line was plated in 6-well plates allowed for clone formation. (d) The clones were stained with crystal violet (0.5% w/v) and photographed. (e) Average results of three independent experiments were plotted. ^*^, p<0.05, ^* *^, p<0.01.

**Figure 7:**
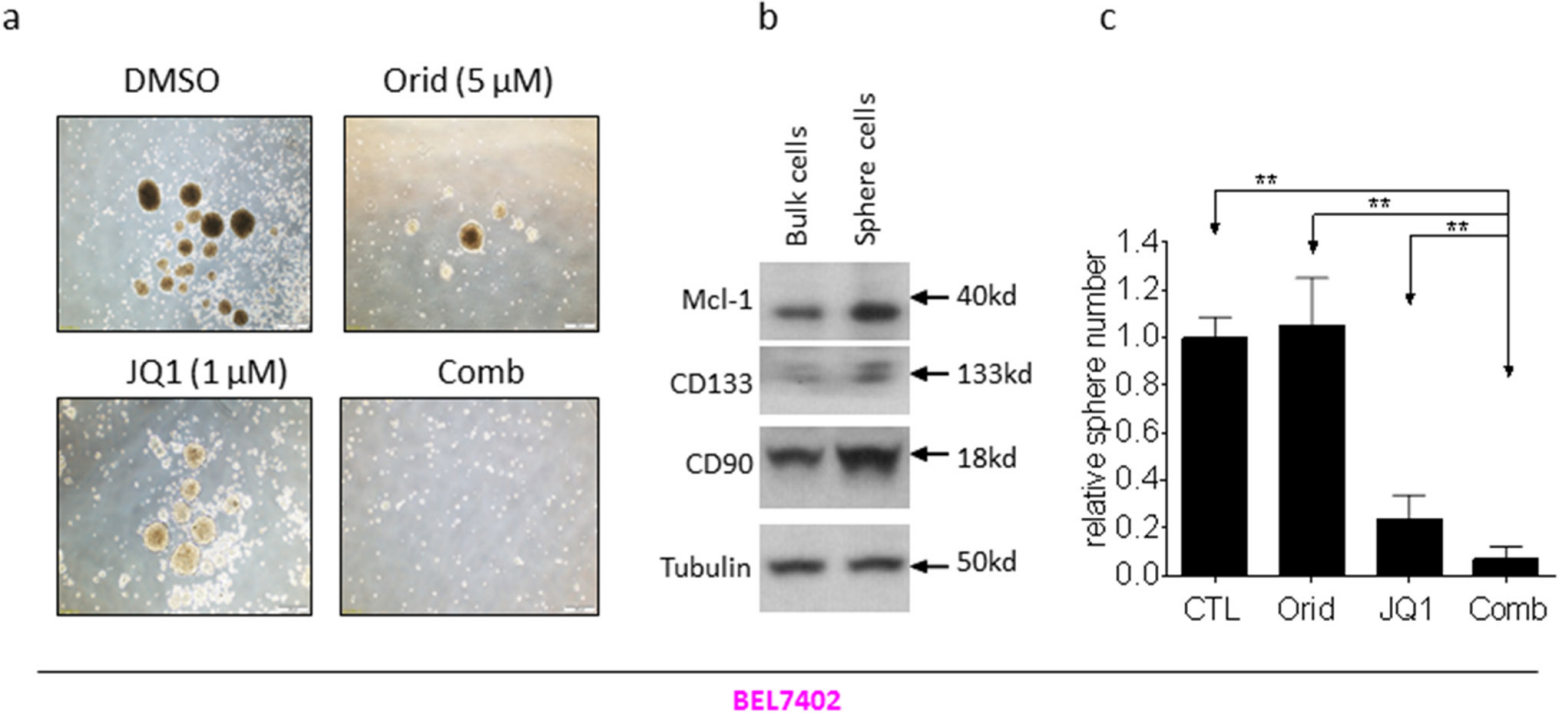
Oridonin significantly enhance JQ1-mediated inhibition of BEL7402 CSC-like cells growth HCC BEL7402 cell line was plated in stem cell conditioned culture system allowed for sphere forming. The number of spheres was counted under a microscope and photographed. **(a)** One representative figure was shown for each treatment. **(b)** The expression of CD90, CD133 and Mcl-1 in both bulk cells and sphere cells were examined examined by western blotting analysis. Tubulin was used as a loading control. **(c)** Average results of three independent experiments were plotted. ^*^, p<0.05, ^* *^, p<0.01.

Sphere formation is used as a surrogate *in vitro* CSC assay [15, 16]. We next investigated whether the combination had effect on HCC cells to form spheres. We grew spheres from two HCC cell lines with serum-free medium under low-attachment condition. Decent numbers of spheres were formed in both HCCLM3 and BEL7402 cell lines (Figure 6a, Figure 7a). Western blotting analysis showed that the spheres cells displayed characteristics of HCC CSC by expressing higher levels of CD90 and CD133, as well as Mcl-1 (Figure 6b, Figure 7b). Although the cells treated by JQ1 still formed spheres with decent size and nature, the spheres number is smaller than that treated by DMSO. Oridonin treatment also modestly reduced the number and the diameter of HCC spheres. Impressively, the combination completely abolished the ability of HCC cells to form spheres in both cell lines (Figure 6a, 6c and Figure 7a, 7c).

CSCs more readily form clones in cell culture [17, 18]. We next investigated whether the combination had effect on clone formation. HCC cells were cultured in 6-well plates and treated with Oridonin alone, JQ1 alone or their combination as indicated. Appreciable number of clones was formed in wells treated by DMSO for 12 days in HCCLM3 cell line. Treatment with JQ1 or Oridonin alone inhibited clone number by 34% and 43%, respectively. In contrast, treatment by the combination almost completely eliminated clone formation (Figure 6d, 6e).

These results suggest that the combination have much more profound inhibitory effect on the growth of HCC CSCs.

## DISCUSSION

BET inhibitors have emerged as a novel class of drug candidates. Preclinical studies with BET inhibitors demonstrated attractive anticancer activities through suppression of aberrantly activated oncogenic factors, such as cMyc, p27, cdt1 in hematological malignancies [2–4]. Clinical studies also have shown favorable response in hematological malignancies [19]. However, the bioactivity of these new agents in solid cancers is generally limited [6–8]. BET inhibitors did not induce cancer remission in tumor-bearing mice in most preclinical tumor models, and there is no obvious clinical response within solid tumors. Accordingly, recent studies have shifted attention on exploration of combinational strategies for overcoming resistance of BET inhibitors in diverse types of cancers. For instance, Ma et al found that MAPK pathway inhibitor trametinib synergistically promoted the antitumor activity of JQ1 in colorectal cancer cells [20]. Paoluzzi et al showed that BRAF inhibitors could be used to overcome JQ1 BRAF-mutant resistance in melanoma cells [7]. We here reported another strategy to improve the anticancer activity of BET inhibitors. In this strategy, we found that Oridonin could be used to override the apoptosis resistance to BET inhibitors in HCC cells. This strategy is special because Oridonin as a conventional anticancer medicine has long been used safely in diverse clinical settings in China [21–23]. This strategy therefore has translational significance for the potential use of BET inhibitors in treatment of HCC.

A main resistance mechanism underlying JQ1-resistance is the ineffectiveness of apoptosis induction in solid tumor cells [6–10]. In this study, we found that Oridonin overcame JQ1-resistance through significantly enhanced apoptosis induction. We reached this conclusion by multiple lines of evidence. They include: (a) the combination treatment significantly led to more HCC cells positively conjugated with Annxin-V, a surface biomarker of apoptosis induction; (b) the combination triggered stronger activation of apoptosis signaling than either single-agent; (c) the combination triggered more pro-apoptotic factor cytochrome c release than either single-agent; and (d) HCC cells could be rescued from the combination-induced death by a caspase-9 inhibitor. Apoptosis is a predominant mechanism by which various therapies kill cancer cells [13, 14]. These findings thus suggest that simultaneously application of BET inhibitors and Oridonin may be more efficiently in killing HCC cells, which suggest that this strategy could be used to improve efficacy, to prevent tumor relapse and to inhibit metastasis in the treatment of HCC.

Apoptosis is executed by caspases, a group of enzymes with proteolytic function [24]. The activity of caspases is strictly regulated by a number of proteins at different phase of apoptosis. The balance of pro-apoptotic and anti-apoptotic Bcl-2 members controls the mitochondrial membrane potential and caspase activation during initiation of apoptosis. The reduction of anti-apoptotic and/or increase of pro-apoptotic Bcl-2 proteins will cause damage of mitochondrial membrane integrity. This in turn leads to cytochrome c released to cytoplasm from mitochondrial intermembrane space where the initiator pro-caspase-9 and effector pro-caspase-3 are cleaved and activated [24]. Moreover, the activity of caspases-9, -3 can be inhibited by inhibitor of apoptosis (IAP) during the execution phase of apoptosis [25, 26]. Our data showed that Oridonin distinctly reduced the expression of Bcl-2, Mcl-1 and xIAP. These data suggest that the enhancement of JQ1-triggered apoptosis by Oridonin is most likely mediated by the suppression of multiple anti-apoptotic proteins.

Cancer stem cells (CSCs) represent the malignant subpopulations that initiate HCC development and mediate tumor relapse and metastasis [27–29]. Our data showed that the combination more effectively inhibited the growth of CSCs. This suggests that the combination treatment has the potential to be used for prevention of tumor relapse and metastasis in HCC treatment.

## MATERIALS AND METHODS

### Cell lines and compound preparation

The HCC cell lines HCCLM3, BEL7402, MHCC97H and SMMC7721 were obtained from the China Center for Type Culture Collection (Wuhan, China) and maintained in high-glucose DMEM (HyClone/Thermo Fisher Scientific, Beijing, China) supplemented with 10% heat-inactivated fetal bovine serum (Hangzhou Sijiqing Biological Engineering Materials Co., Ltd, Hangzhou, China). JQ1 was kindly gifted by Professor James Bradner (Harvard Medical School). JQ1 was dissolved in Dimethyl sulfoxide (DMSO) at a stock concentration of 10 mmol/L and stored at −20°C.

### CCK-8 assay

Cell viability was measured by a WST-8 [2-(2-methoxy-4-nitrophenyl)-3-(4-nitrophenyl)-5-(2,4-disulfophenyl)-2H-tetrazolium, monosodium salt] assay (Dojindo Molecular Technologies, Inc., Rockville, MD). Cells were plated in 96-well plates and cultured overnight to allow cells to attach, and then the drug was added at indicated concentrations for 120 hours. WST-8 dye was added to each well to final concentration (v/v) of 10% and incubated for an additional 2 hours, and the absorbance at 450 nm was measured in a microplate reader (Molecular Devices, Sunnyvale, CA). Cell viability was evaluated as the ratio of the absorbance of the drug-treated samples to that of the DMSO-treated control and analyzed by Prism 6 software. All experiments were carried out in triplicate. The percentages of absorbance relative to those of untreated control samples were plotted as a function of drug concentration (log scale). Inhibition of cell viability was measured by percentage of viable cells relative to the control: inhibition% = 1-100% × ODT / ODC, where ODT is the average OD value of the treated samples and ODC is the average OD value of the control samples.

### Flow cytometry and cell viability assays

Apoptosis analysis was done using an Annexin V/propidium iodide (PI) apoptosis detection kit (BD Biosciences, Shanghai, China) by flow cytometry according to the manufacturer's instructions. Cells positively stained with Annexin V were counted as apoptotic cells. Cell viability was examined by trypan blue staining and manually counting under microscopy. Cells positively stained or obvious morphologically changed was considered inviable cells, otherwise considered as viable cells.

### Cell fractionation

HCC cells were treated as indicated, collected, washed with PBS and suspended in 5 volumes of chilled buffer A (250 mM sucrose, 20 mM HEPES, 10 mM KCl, 1.5 mM MgCl2, 1 mM EDTA, 1 mM EGTA, 1 mM DL-dithiothreitol [DTT], 17 μg/mL phenylmethylsulfonyl fluoride [PMSF], 8 μg/mL aprotinin and 2 μg/mL leupeptin [pH 7.4]) on ice for 15 min. Cell fractionation was performed using the homogenization method. Briefly, cells were homogenized using an ice-cold cylinder cell homogenizer (20-25 strokes). Homogenized cell lysates were separated by centrifugation at 750 g for 10 min, and the supernatants were further centrifuged at 10,000 g for 20 min. The remaining supernatant was used as the cytosolic fraction and subjected to western blot analysis.

### Western blotting

Cells were lysed using radioimmunoprecipitation (RIPA) assay lysis buffer (PBS containing 1% NP40, 0.5% Na-deoxycholate, and 0.1% SDS) supplemented with 1 μmol/L phenylmethylsulfonyl fluoride and 1 protease inhibitor cocktail tablet per 10 mL on ice for 20 min, and lysates protein concentration were determined using the Bio-Rad protein assay kit according to the manufacturer’s instructions. Proteins were electrophoresed onto 4-20% SDS-PAGE gels (Invitrogen, Carlsbad, CA, USA) and transferred onto polyvinylidene difluoride membranes. Following blocking in 5% milk, the membranes were incubated with a specific primary antibody, washed, and incubated with horseradish peroxidase-linked secondary antibody (GE Healthcare, Beijing, China). Signals were visualized with chemiluminescent horseradish peroxidase antibody detection reagent (Denville Scientific, Guangzhou, China).

The antibodies used were as follows: Bcl-2 (C-2) (sc-7382), Mcl-1 (S-19)(sc-819), Bak (G-23) (sc-832), caspase-9 (LAP 96-2-22)(sc-56077), caspase-3 (H-277) (sc-7148) were purchased from Santa Cruz Biotechnology (Shanghai, China). xIAP (2042), PARP (9542), Cleaved Caspase-9 (Asp330) (9501), cytochrome c (4272), COX IV (4850) and Tubulin (2144) were purchased from Cellular Signaling (Shanghai, China). cIAP-1/2 (ab25939) were purchased from Abcam (Shanghai, China).

### Sphere culture of HCC cells

The dissociated HCC cells (1×3,000 per well) from HCCLM3 and BEL7402 were seeded on 6-well plates and cultured in ultra-low attachment culture plates (Corning) with DMEM-F12 supplemented with 20 ng/μl hEGF (Gibco), 20 ng/μl bFGF (Gibco), 1x B27 (Invitrogen), 1x Insulin-Transferrin-Selenium A (Invitrogen) and 100 U/100 μg/ml penicillin-streptomycin; serum free medium [16]. Half of the medium was replaced with fresh medium every 3 days. After 1 week of cultivation, the number of spheres was counted under a microscope and photographed.

### Clonogenic assays

For clonogenic assays, 1,500 cells were seeded into 6-well dishes in 5 mL of medium, treated as indicated, and maintained for 12 days at 37°C in a 5% CO_2_ incubator. Cells were then washed with drug-free medium, stained with 0.01% (w/v) crystal violet, and cell colonies (> 50 cells) were counted at 14 days post-treatment. The assays were performed in duplicate with at least three different repeats per treatment.

### Statistical analyses

All data are displayed as the mean ± SEM unless specified otherwise. T-test was used to evaluate statistical significance (p < 0.05 was deemed significant).
